# *NSD3::NUTM1* Fusion Sarcoma Mimicking Malignant Peripheral Nerve Sheath Tumor with Prolonged Survival

**DOI:** 10.3390/biomedicines12081709

**Published:** 2024-08-01

**Authors:** Jing Di, Ali M. Alhaidary, Chi Wang, Jinge Liu, Sainan Wei, Joseph Valentino, Therese J. Bocklage

**Affiliations:** 1 Department of Pathology & Laboratory Medicine, University of Kentucky, Lexington, KY 40536, USA; ali.alhaidary@uky.edu (A.M.A.); sainan.wei@uky.edu (S.W.); 2 Division of Cancer Biostatistics, Department of Internal Medicine, College of Medicine, University of Kentucky, Lexington, KY 40536, USA; chi.wang@uky.edu; 3 Biostatistics and Bioinformatics Shared Resource Facility, Markey Cancer Center, University of Kentucky, Lexington, KY 40536, USA; jinge.liu@uky.edu; 4 Head, Neck & Respiratory Clinic, Markey Cancer Center, University of Kentucky, Lexington, KY 40536, USA; joseph.valentino@uky.edu

**Keywords:** malignant peripheral nerve sheath tumor, *NSD3::NUTM1* fusion, RNA sequencing, neurofibroma, NUT sarcoma

## Abstract

Nuclear Protein in Testis (NUT)-rearranged tumors comprise predominantly NUT carcinoma but also include certain lymphomas, leukemias, skin appendage tumors, and sarcomas. Although histologically diverse, all are genetically identified by oncogenic rearrangement in the NUTM1 gene. Many fusion partners occur, and NSD3 is NUT carcinoma’s third most common partner. Herein, we present a case of a 26-year-old man with an *NSD3::NUTM1* fusion sarcoma. The patient presented at the age of 13 months with a scalp nodule. Over the next 24 years, he experienced five local recurrences and ultimately expired of a rapidly progressive recurrence. His treatment included surgical resections, radiation, and various chemotherapies. Deceptively, the clinical presentation and histopathology aligned with a malignant peripheral nerve sheath tumor, a diagnosis rendered at initial resection with concurrence by a national soft tissue tumor expert. The patient’s exceptionally long survival could be due to *NSD3* as the fusion partner, aided by the initial small tumor size and young patient age. Thus, this case expands NUT fusion sarcomas’ histologic and immunohistochemical profile to include mimicking a malignant peripheral nerve sheath tumor (MPNST). Additionally, it indicates that the *NSD3::NUTM1* fusion can drive sarcoma genesis.

## 1. Introduction

Here, we present a case of a 26-year-old man with a history of a multiply recurrent malignant peripheral nerve sheath tumor (MPNST) whose tumor was found to harbor an oncogenic *NSD3::NUTM1* fusion. NUT fusion tumors comprise a diversity of tumor types that include NUT carcinoma and specific types of lymphomas/leukemias, skin appendage tumors, and sarcomas [1]. Uniting these tumors is an oncogenic *NUTM1* rearrangement with various fusion partners. NUT carcinoma is the most well characterized of the NUT fusion tumors, a poorly differentiated carcinoma considered a subtype of highly aggressive squamous cell carcinoma, previously known as NUT midline carcinoma [2,3]. NUT carcinomas exhibit histologic features of a malignant epithelial tumor, including the expression of epithelial antigens. The most common fusion gene partners in NUT carcinoma are *BRD4*, *BRD3*, and *NSD3*. The *NSD3::NUTM1* fusion has not been previously reported in a sarcoma. Moreover, an *NUTM1* fusion has not been previously reported in a tumor diagnostically consistent with MPNST. *NSD3::NUTM1* fusions in sarcomas and their poorly understood biological implications necessitate a deeper examination of their oncogenic activity and clinical significance [1,2,3,4]. Different fusion partners in NUT carcinomas can significantly influence tumor behavior and patient prognoses, underscoring the need for identifying specific fusion types to inform clinical practice and potential therapeutic targets [5].

Our patient’s tumor, based on its morphology and immunohistochemistry expression profiling, aligned with a diagnosis of MPNST initially. MPNST is a rare and aggressive soft tissue sarcoma that may arise sporadically, associated with neurofibromatosis type 1, or secondary to radiation [6]. Genetically, MPNSTs are characterized by frequent inactivating mutations in *NF1, CDKN2A/2B*, and *PRC2* core components’ (*EED* or *SUZ12*) three pathways, resulting in complete loss of function [7,8]. Traditional sarcoma classification, dominated by aggregating clinical findings, histologic features, and the immunohistochemical expression profile, generally enables an accurate diagnosis. However, our patient’s case, which fit the traditional classification profile for MPNST but instead harbored an unexpected *NSD3::NUTM1* fusion, provides an example of discordance between histology and genetics. Thus, our case underscores the value of comprehensive genomic profiling in managing sarcomas to identify potential therapeutic targets and appropriately classify tumors.

## 2. Case Presentation

### 2.1. Clinical History

A 26-year-old male with a history of MPNST presented with two recurrent scalp and neck masses measuring 4 × 3 cm located in the right occipital scalp and 7 × 5 cm located in the right neck, respectively. The patient’s disease course was exceptionally extended (see Table 1 for timeline). He was first diagnosed at age 13 months, presenting with a posterior scalp mass. Over the next 25 years, he experienced five local recurrences. His first recurrence occurred at age six and was managed with surgical resection followed by external beam radiation therapy (EBRT). Subsequent recurrences at ages 12 and 20 were treated similarly with resections and additional EBRT. His participation in the COG D9902 clinical trial offered a period of remission. However, at age 22, imaging revealed a fourth recurrence, leading to wide local re-excision, and included the finding of a plexiform neurofibroma, with the absence of other classic neurofibromatosis signs. At 23, a dull ache in his scalp and a hypermetabolic scalp nodule hinted at yet another recurrence, which was confirmed via fine needle aspiration. Subsequent wide local re-excision revealed a recurrent sarcoma. At 26, during his sixth recurrence, rapid and deep local tumor progression was evident through serial imaging. Complications escalated with the confirmation of leptomeningeal carcinomatosis and skull base metastases via MRI, which corresponded with clinical symptoms of hydrocephalus, hyponatremia, and respiratory challenges. His condition deteriorated, marked by pain, numbness, neck swelling, diplopia, and weakness in his lower extremities. After personal reflection and consultation with family and caregivers, the patient declined additional aggressive therapy and entered hospice care. He died of tumor complications 25 years after his initial presentation at age 13 months.

### 2.2. Morphologic and Immunohistochemical Overview of Tumor Recurrences

**Figure 1:**
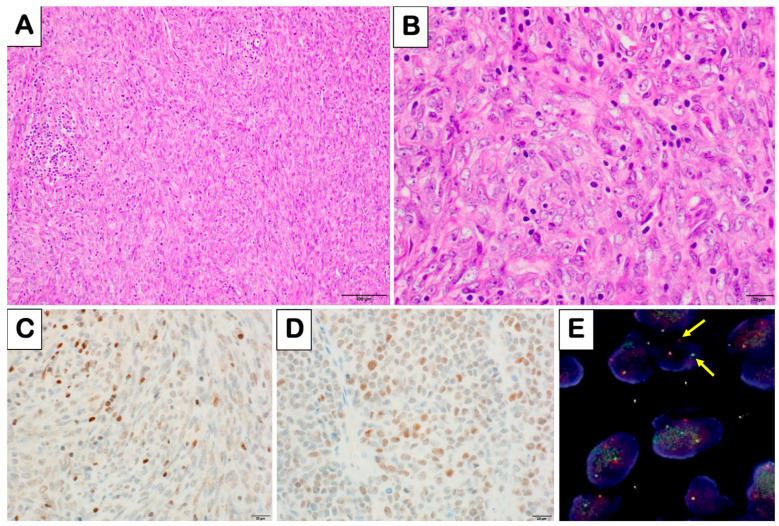
Histological, immunohistochemical, and genetic analyses of the recurrence #2 tumor sample. (**A**,**B**): Hematoxylin and eosin-stained sections displaying epithelioid tumor cells at two magnifications: 200× in panel A and 600× in panel B, highlighting cellular morphology. (**C**): Immunohistochemical staining for H3K27me3 at 600× magnification, showing weak to absent expression in most tumor cells. (**D**): Immunohistochemical staining for NUT protein at 600× magnification, illustrating NUT protein expression in tumor nuclei. (**E**): Fluorescence in situ hybridization (FISH) results at 600× magnification demonstrating the separation of 5′ and 3′ NUTM1 gene probes, with yellow arrows indicative of NUTM1 gene rearrangement in tumor cells.

The initial surgical pathology report describing the patient’s tumor at age 13 months was not available. At his first recurrence at age 6, the immunohistochemical analysis showed neurofilament, collagen IV, Leu 7, and vimentin expression, with negative results for S-100, EMA, CD99, desmin, and keratin. At the second recurrence at age 12, the tumor comprised a mixture of plump and spindle epithelial cells, with a mitotic count of 5 per mm^2^ (Figure 1A,B). The third recurrence at age 20 revealed a similar mix of predominantly plump epithelial and spindle cells, with a mitotic count of 4 per mm^2^.

**Figure 2:**
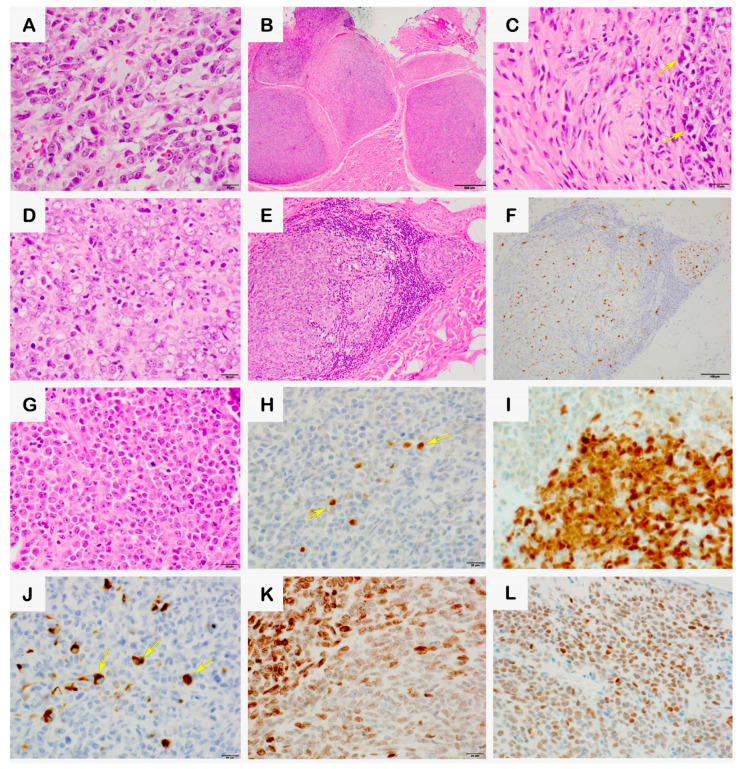
Morphological and immunohistochemical characterization features of the recurrence #4, #5, and #6 tumors with adjacent plexiform neurofibroma-like nerve bundles. (**A**–**C**): Hematoxylin and eosin-stained sections of the recurrence #4 tumor: (**A**): Display of plump epithelial tumor cells at 600× magnification, emphasizing their dense, eosinophilic cytoplasm. (**B**): The visualization of adjacent nerve bundles, highlighting their structural alignment and density at 100× magnification. (**C**): The depiction of atypical cells within the nerve bundles, noted for their irregular morphology and nuclear features (yellow arrows), at 600× magnification. (**D**–**F**): Hematoxylin and eosin-stained sections of the recurrence #5 tumor: (**D**): Presentation of plump tumor cells at 600× magnification, similar in appearance to those in recurrence #4. (**E**): Illustration of adjacent neurofibroma-like tumor cells, showing their integration with the nerve bundles at 200× magnification. (**F**): Immunohistochemical staining showing the patchy positive expression of S-100, indicating variable antigen expression across the tumor cells at 200× magnification. (**G**–**L**): Hematoxylin and eosin- and immunohistochemical-stained sections of the recurrence #6 tumor at 600× magnification—(**G**): Hematoxylin and eosin-stained section, showing the heterogeneous cellular architecture of the tumor. (**H**): Immunohistochemical staining for the neurofilament, focally positive, highlighting the presence of nerve cell markers in tumor cells (yellow arrows). (**I**): Immunohistochemical staining for S-100, partially positive, demonstrating the expression of this neural crest marker in a subset of tumor cells. (**J**): Immunohistochemical staining for SOX-10, focally positive, with yellow arrows indicating the presence of this transcription factor involved in neural crest development. (**K**): Immunohistochemical staining for H3K27me3, showing weak to moderate expression and providing insights into the epigenetic landscape of the tumor. (**L**): Immunohistochemical staining for NUT protein, showing expression consistent with the *NSD3::NUTM1* fusion in the tumor.

The patient’s fourth recurrence at age 22 featured large cells with vesicular nuclei and an increased mitotic count of 15 per mm^2^ (Figure 2A). Additionally, adjacent plexiform neurofibroma-like nerve bundles were observed with intermingled atypical cells (Figure 2B,C). The fifth recurrence at age 23 involved small round cells and patchy rhabdoid cells (Figure 2D), a mitotic count of 12 per mm^2^, and a negative expression of S-100, SOX10, HMB45, and Melan-A. Adjacent neurofibroma-like nerve bundles were noted, exhibiting patchy S-100 expression (Figure 2E,F). The sixth recurrence at age 26 revealed a heterogeneous mixture of small round blue cells and rhabdoid cells set in a myxoid background (Figure 2G), with a mitotic count of 13 per mm^2^ and less than 50% geographic necrosis. The immunohistochemical analysis was positive for vimentin, focally positive for the neurofilament (Figure 2H), and variably positive for S-100 and SOX10 (Figure 2I,J), with INI1 retained.

### 2.3. Advanced Immunohistochemical Analysis

Immunohistochemistry for NUT protein and H3K27me3 was performed on formalin-fixed paraffin-embedded tissues from recurrences #2 to #6 at Mayo Clinic Laboratories with interpretation in the UK. The nuclear expression of NUT protein and loss of H3K27me3 were assessed relative to internal controls. The recurrence #2 tumor showed the patchy expression of NUT and patchy loss of H3K27me3 expression (Figure 1C,D). The recurrence #6 tumor showed the expression of H3K27me3 and NUT (Figure 2K,L), demonstrating a complex and evolving immunophenotypic profile.

### 2.4. Genetic Testing

#### 2.4.1. Karyotype (Performed on Recurrence #6)

In total, 75% of metaphase cells examined showed chromosomal abnormalities with two translocations, t(7;12)(q22; q24.3) and t(8;15)(p11.2; q11.2).

#### 2.4.2. Fluorescence In Situ Hybridization (FISH) (Performed on Recurrences #2, #5, and #6)

To detect the presence of *NUTM1* rearrangement, a break-apart probe set (NUTM1 Break Apart DNA probes; NUTM1BA-20-GROR, Empire Genomics) was used, which targets sequences flanking the NUM1 gene (CHR15: 34343314-34357736). Formalin-fixed paraffin-embedded tissue sections were prepared, and the probes were applied and allowed to hybridize overnight. Post-hybridization washes removed unbound probes, and DAPI was used for counterstaining. Under a fluorescence microscope, the separation of 5′ and 3′ probes indicated a break within the NUTM1 gene. This study scored 40 cells per sample, revealing that approximately 70% of the cells exhibited NUTM1 rearrangement (Figure 1E).

#### 2.4.3. Germline Assessment (Performed on Recurrence #5)

Germline assessment was conducted using Invitae genetic testing (Invitae, San Francisco, CA, USA) and was negative for NF1 alterations.

#### 2.4.4. Next-Generation Sequencing (NGS)

RNA-seq analyses were conducted on recurrence #5 and #6 tumor tissues using two NGS platforms (CARIS Life Sciences, Phoenix, AZ, USA, and AVATAR, ORIEN Oncology Research Information Exchange Network, Hudson, FL, USA). An *NSD3::NUTM1* fusion was found at two distinct breakpoints. In the recurrence #5 sample, the fusion occurred at the exon 7 (breakpoint at chromosome 8 position 38318895) and exon 3 (at chromosome 15 position 38318895) splice site. In the recurrence #6 sample, the fusion was identified at the exon 9 (chromosome 8 position 38176413) and exon 3 (chromosome 15 position 34640170) splice site. The transcript IDs corresponding to these findings are NM_017778.2 and NM_175741.2. Unclassified translocations *NT5C2::SNRPD2* and *SOX11::ZKSCAN1* in recurrence #5 and ARHGAP42::CNTN5 in recurrence #6 were identified.

**Figure 3:**
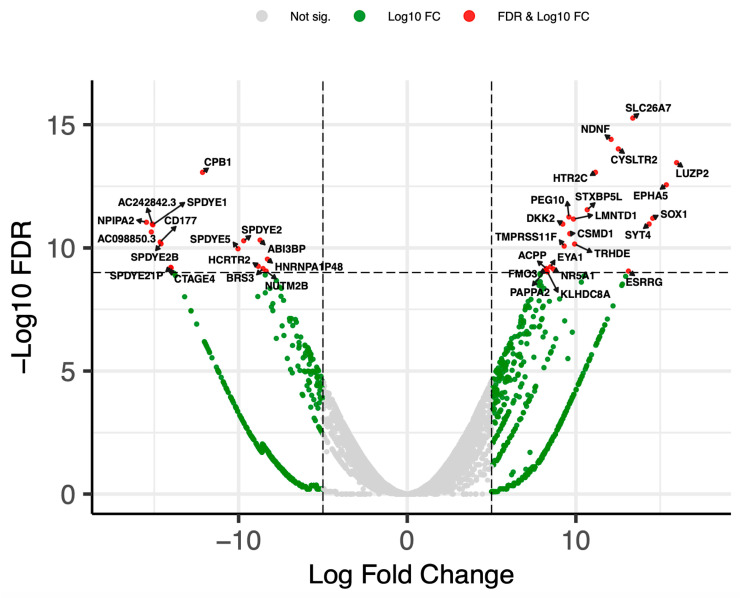
A volcano plot displaying the differential gene expression recurrence #5 and #6 tumor samples, where the x-axis represents log2 fold change and the y-axis indicates –log10 (FDR), highlighting genes significantly upregulated (to the right) and downregulated (to the left) with the top 20 genes marked with the red color and labeled. Total = 18,901 variables.

Gene expression was examined with Gene Set Enrichment Analysis (GSEA 4.3.2) software to assess over 2000 differentially expressed genes (DEGs), which were then ranked and visualized (Figure 3). The top 40 DEGs were analyzed, revealing a spectrum of gene upregulation and downregulation differences between the recurrence #5 and #6 tumors (Supplementary Figure S1 and Supplementary Table S1).

#### 2.4.5. Methylation Profiling Testing

Genomic DNA was extracted from the recurrence #6 formalin-fixed paraffin-embedded (FFPE) tissue sections. Macro-dissection was performed to enrich tumor content, and DNA was bisulfite-converted and analyzed using the Infinium Methylation EPIC kit on an iScan reader. Tumor classification and molecular profiling were conducted using established classifiers and an in-house pipeline. The integrative diagnosis was determined by combining methylation profiling, next-generation sequencing (NGS) results, histopathology, clinical history, and the relevant literature. The Laboratory of Pathology at NCI developed and performed the tests according to CLIA requirements. The material available was deemed adequate for molecular evaluation, with an estimated 70% fraction of viable lesional cells. Despite using versions 11b6 and 12b6 of the Heidelberg and the NCI/Bethesda classifier, no matching methylation class was identified.

## 3. Discussion

This novel case of a 26-year-old man with a long-standing diagnosis of MPNST but who exhibited an *NSD3::NUTM1* fusion as early as his second recurrence at age 12 years presents a complex diagnostic and therapeutic challenge. Diagnosing sporadic MPNSTs is challenging due to genetic and immunohistochemical heterogeneity, often necessitating a diagnosis by exclusion [6]. Histologically, the tumors can consist of spindle, mixed spindle and epithelioid, or entirely epithelioid cells, and cellular pleomorphism varies [7]. Some tumors exhibit biphasic differentiation with epithelial components, including well-formed glands [7]. Immunohistochemical antibodies to S-100 and SOX-10 are variably expressed [8]. The patient’s first recurrence expressed the neurofilament, Leu 7, and vimentin but lacked S-100 protein, EMA, and keratin. This supported an interpretation of neuronal differentiation and, given the tumor’s histologic features and location in soft tissue, was consistent with a diagnosis of MPNST. Moreover, in recurrence # 4 and #5, the tumor displayed features of involvement of a plexiform neurofibroma, a cancer found almost exclusively in patients with neurofibromatosis type 1 [9]. However, there was no clinical evidence of NF1 nor a germline or tumor mutation in the *NF1* gene.

Notably, patchy loss of H3K27me3 expression in #2 and #6 recurrences suggests a perturbance of regular H3K27me3 expression but not to the point of complete loss as is notable in ~80% of MPNSTs [6,7]. Morphologically, the tumors showed consistent features over two decades, comprising sheets and lobules of monotonous, plump, epithelioid cells ± plump spindle cells with abundant eosinophilic cytoplasm. The final recurrences, however, also featured rhabdoid cell morphology, small round epithelioid cells, and a significantly elevated mitotic rate.

The *NSD3::NUTM1* fusion is reported exclusively in NUT carcinoma, an aggressive cancer typically characterized by epithelial differentiation and considered a subtype of squamous cell carcinoma [6,7]. The presence of this specific *NUTM1* fusion in what otherwise met histologic and IHC diagnostic criteria for an MPNST required a re-examination of the MPNST diagnosis and consideration of a diagnosis of either NUT carcinoma (albeit in an unusual location) or a NUT sarcoma with phenotypic peripheral nerve sheath cell differentiation. It is unlikely that the patient had a pre-existing typical MPNST that then underwent random molecular alterations that included a NUT fusion because somatic genetic evidence for MPNST in the patient’s tumor is lacking. *NF1* was neither mutated nor lost in cancer, germline sequencing was negative for an *NF1* mutation, and the patient’s karyotype lacked complex aneuploidy typical of MPNST. On the sequencing analysis, the patient’s recurrence #6 showed a low tumor mutation burden of 2 mut/Mb and a low level of loss of heterozygosity at 1% of tested genomic segments, also atypical for MPNST [6]. Furthermore, the NUT fusion was established by age 12 years (recurrence #2), suggesting that the NUT fusion was present probably at tumor initiation and that additional molecular alterations accruing at recurrence #6 accelerated tumor aggressiveness.

NSD3, a histone lysine methyltransferase from the NSD family, modulates gene expression via histone marks and interacts with BRD4’s ET domain in the NSD3::NUTM1 fusion process, critical for the function of the BRD4-NUTM1 oncoprotein and potentially influencing gene transcription in neuroectodermal (NC) cells by regulating H3K36 methylation or macroH2A1 interaction [4,10,11,12]. The *NSD3::NUTM1* fusion protein is necessary and sufficient for maintaining the undifferentiated state in NC cells [12]. Notably, patients with NUT carcinoma in the head and neck region and who exhibit the *NSD3::NUTM1* fusion tend to have a notably improved prognosis compared to the typical NUT carcinoma median survival of 6.5 months, with a median overall survival of 36.5 months [13]. In an extraordinary case from a recent series, the patient survived for an impressive duration of 14 years post-positive identification of the NUTM1 break-apart in the tumor [14]. Thus, there is precedence for prolonged survival in patients whose NUT fusion tumors harbor *NSD3::NUTM1* fusions.

The gene set enrichment analysis performed on recurrence #5 and #6 showed over 2000 differentially expressed genes (DEGs). Repeated bouts of radiation therapy and chemotherapy could have affected the gene expression and selection of subclones. Notably, four genes involved in neural development or function (*SOX1*, *NDNF*, *HTR2C, SYT4*) were significantly overexpressed in recurrence #6 in keeping with persistent aberrant differentiation toward a neural-like phenotype [15]. This patient was not treated with Bromodomain and extra-terminal (BET) inhibitors, which may potentially target NSD3-driven tumorigenesis [16,17]. The number of NSD3 inhibitors available for cancer therapy is currently limited, and no drugs targeting NSD3 are available on the market [4]. Future research should focus on the development of specific inhibitors to target NSD3 fusion proteins in order to improve therapeutic outcomes for patients with such rare sarcomas [16].

The limitations of this study are rooted in its nature as a case report. Whether our findings can be applied to other MPNST cases remains unknown. Possibly, sarcomas that appear compatible with a diagnosis of MPNST but show NUT fusions could be analogous to *CIC::NUTM1* fusion tumors, in which the CIC fusion protein drives the phenotypic and methylation profile of the cancer to align with classic *CIC*-fused sarcomas [18].

In summary, our patient’s tumor is the first “MPNST” to show an *NSD3::NUTM1* fusion, expanding the phenotypic profile of NUT fusion sarcomas. The facility of *NUTM1* fusion tumors to develop into multiple different tumor types, with corresponding corroborating histologic features and immunohistochemical profiles, suggests that it is prudent to include a molecular genetic analysis in cases where a sarcoma such as an MPNST exhibits unusual features. Our case also indicates that the *NSD3* fusion partner can drive carcinogenesis and sarcoma genesis, with the resulting tumor phenotype most likely dependent on the originating cell type.

## Figures and Tables

**Table 1:** Histologic, immunohistochemical, and genetic findings and treatments.

Tumors	H&E Morphology	Mitosis (Count/mm^2^)	Necrosis	Immunohistochemistry	Genetic Tests	Treatment
Initial tumor at 13 months	Not recorded	Not recorded	None	Not recorded	Not performed	Resection
Recurrence #1 at age 6	Not recorded	Not recorded	None	Positive for neurofilament, collagen IV, Leu 7, and vimentin; negative for S-100, EMA CD99, desmin, and keratin	Not performed	Resection and EBRT
Recurrence #2 at age 12	Mix of plump spindled and epithelioid tumor cells in sheets	5	None	Negative for NUT; H3K27me3 weakly patchy positive	Not performed	Resection and EBRT; enrolled in COG D9903
Recurrence #3 at age 20	Mix of plump epithelioid and spindled cells in sheets	4	None	Not performed	Not performed	Resection and XRT 6100 cGy
Recurrence #4 at age 22	Large cells with vesicular nuclei	15	None	Not performed	Not performed	Resection
Recurrence #5 at age 23	Small round cells with patchy rhabdoid cells	12	None	Negative for S-100, SOX10, HMB45, and Melan-A	Negative for *NF1* alterations by Invitae; Whole Genome NGS by CARIS Life Sciences	Resection and expectant management
Recurrence #6 at age 26	Mix of small round blue cells and rhabdoid cells in a myxoid background	13	<50% geographic necrosis	Positive for vimentin; patchy positive for NUT, S-100, and SOX10; INI1 retained; focal positive for neurofilament; patchy positive for H3K27me3	Whole Genome NGS by ORIEN AVATAR	Incomplete resection

## Data Availability

The datasets generated and analyzed during the current study are available from the corresponding author upon reasonable request. Additional data supporting the findings of this study are included within the article and its Supplementary Materials. All relevant data are contained within the manuscript, and high-resolution data are stored securely by the corresponding research institution and are available under specific conditions to researchers who meet the criteria for confidential data access.

## Supplementary Materials

The following supporting information can be downloaded at: https://www.mdpi.com/article/10.3390/biomedicines12081709/s1, Figure S1: The top 40 ranked genes show a range of upregulated and downregulated gene expressions between Recurrence #5 and #6 tumor samples, as indicated by their log FC; Table S1: The top 40 ranked genes’ expression changes (listed in the order according to the degree of up- or downregulation) and potential roles in sarcoma pathogenesis.
