# Supplementary material for: NSD3::NUTM1 Fusion Sarcoma Mimicking Malignant Peripheral Nerve Sheath Tumor with Prolonged Survival
Source: Biomedicines. 2024 Aug 1;12(8):1709. doi: 10.3390/biomedicines12081709 (PMC11351216; doi:10.3390/biomedicines12081709)

Supplemental Table S1. The top 40 ranked genes' expression changes (listed in the order according to the degree of up- or downregulation) and potential roles in sarcoma pathogenesis.

Gene Name	DEG Change	Basic Function	Function in Sarcoma
SLC26A7	UP	Involved in anion transport, especially chloride ions.	Unclear.
NDNF	UP	Neuron-derived neurotrophic factor, involved in neuronal growth and development.	Unclear, may affect tumor microenvironment.
CYSLTR2	UP	Cysteinyl leukotriene receptor 2, involved in inflammatory responses.	Potential involvement in tumor growth and metastasis.
LUZP2	UP	Leucine zipper protein, possibly involved in cytoskeletal organization.	Unclear.
CPB1	Down	Carboxypeptidase B1, involved in protein processing and inflammation.	May influence tumor progression and metastasis.
HTR2C	UP	Serotonin receptor, involved in neurotransmission and various CNS functions.	Possible role in cancer cell proliferation and behavior.
EPHA5	UP	Ephrin receptor, involved in developmental processes and cell-cell interactions.	Ephrin signaling can contribute to tumor growth and progression.
STXBP5L	UP	Syntaxin binding protein, involved in vesicle transport.	Unclear, may affect cellular trafficking.
PEG10	UP	Imprinted gene, plays role in cell proliferation and survival.	Associated with tumor growth and poor prognosis in some cancers.

SOX1	UP	Transcription factor involved in neurogenesis.	Its dysregulation may contribute to sarcoma development.
LMNTD1	UP	Function not well-characterized, possibly involved in nuclear processes.	Unclear.
NPIPA2	Down	Nuclear pore complex interacting protein, function not well-characterized.	Limited information regarding sarcoma.
SYT4	UP	Synaptotagmin, involved in neurotransmitter release.	Unclear.
DKK2	UP	Wnt signaling pathway inhibitor, involved in embryonic development.	May play a role in cancer progression through Wnt signaling.
AC242842.3	Down	Non-coding RNA, function not well-characterized.	Unknown.
SPDYE1	Down	Member of the speedy/ringo family, involved in cell cycle regulation.	Potential involvement in cell cycle dysregulation in tumors.
AC098850.3	Down	Non-coding RNA, function not well-characterized.	Unknown.
CSMD1	Up	Involved in immune response regulation, potentially a tumor suppressor.	May influence tumor immune environment and progression.
ABI3BP	Down	Extracellular matrix protein, involved in cell adhesion and migration.	Possible role in tumor invasion and metastasis.
SPDYE2	Down	Speedy/Ringo cell cycle regulator.	Unclear, could dysregulate cell cycle.
CD177	Down	Neutrophil-specific antigen, involved in immune response.	Unclear, may affect immune interactions.

SPDYE2B	Down	Similar to SPDYE2, involved in cell cycle regulation.	Potential involvement in sarcoma cell proliferation.
TRHDE	UP	Thyrotropin-releasing hormone-degrading enzyme.	Unknown.
TMPRSS11F	UP	Serine protease, involved in proteolytic processes.	May play a role in tumor invasion and metastasis.
SPDYE5	Down	Similar to SPDYE1 and SPDYE2, involved in cell cycle regulation.	Unclear, could affect cell cycle and growth in sarcoma cells.
HNRNPA1P48	Down	Heterogeneous nuclear ribonucleoprotein, involved in RNA processing.	Unclear.
HCRTR2	Down	Hypocretin (orexin) receptor 2, involved in sleep regulation and appetite control.	Unclear, may affect tumor microenvironment.
EYA1	UP	Transcriptional coactivator and phosphatase, involved in organ development.	Unclear.
SPDYE21P	Down	Likely involved in cell cycle regulation, similar to other SPDYE family members.	Unclear, may dysregulate cell cycle.
CTAGE4	Down	Function not well-characterized, possibly involved in cellular transport.	Unknown.
BRS3	Down	Bombesin-like receptor, involved in regulating pituitary function and appetite.	Unknown.
ACPP	UP	Acid phosphatase, prostate, involved in dephosphorylation processes.	May influence tumor microenvironment and metastasis.
NR5A1	UP	Nuclear receptor, involved in steroidogenesis and reproductive system development.	Unclear, possible role in hormonal regulation.

FMO3	UP	Flavin-containing monooxygenase, involved in xenobiotic metabolism.	Unknown.
PAPPA2	UP	Pregnancy-associated plasma protein A2, involved in insulin-like growth factor signaling.	May contribute to tumor growth and progression.
ESRRG	UP	Estrogen-related receptor gamma, involved in energy metabolism and mitochondrial function.	Role in sarcoma unclear, may influence cellular metabolism.
NUTM2B	Down	Member of the NUT midline carcinoma family, involved in transcriptional regulation.	Associated with a rare type of sarcoma called NUT midline carcinoma.
KLHDC8A	UP	Kelch domain containing protein, function not well-characterized.	Unclear.
OR2A1	Down	Olfactory receptor, function primarily in sense of smell.	Unknown.
NPNT	UP	Nephronectin, involved in cell adhesion and migration, particularly in kidney development.	May play a role in tumor cell adhesion and metastasis.

Supplemental Figure S1. The top 40 ranked genes show a range of upregulated and downregulated gene expressions between Recurrence #5 and #6 tumor samples, as indicated by their logFC.

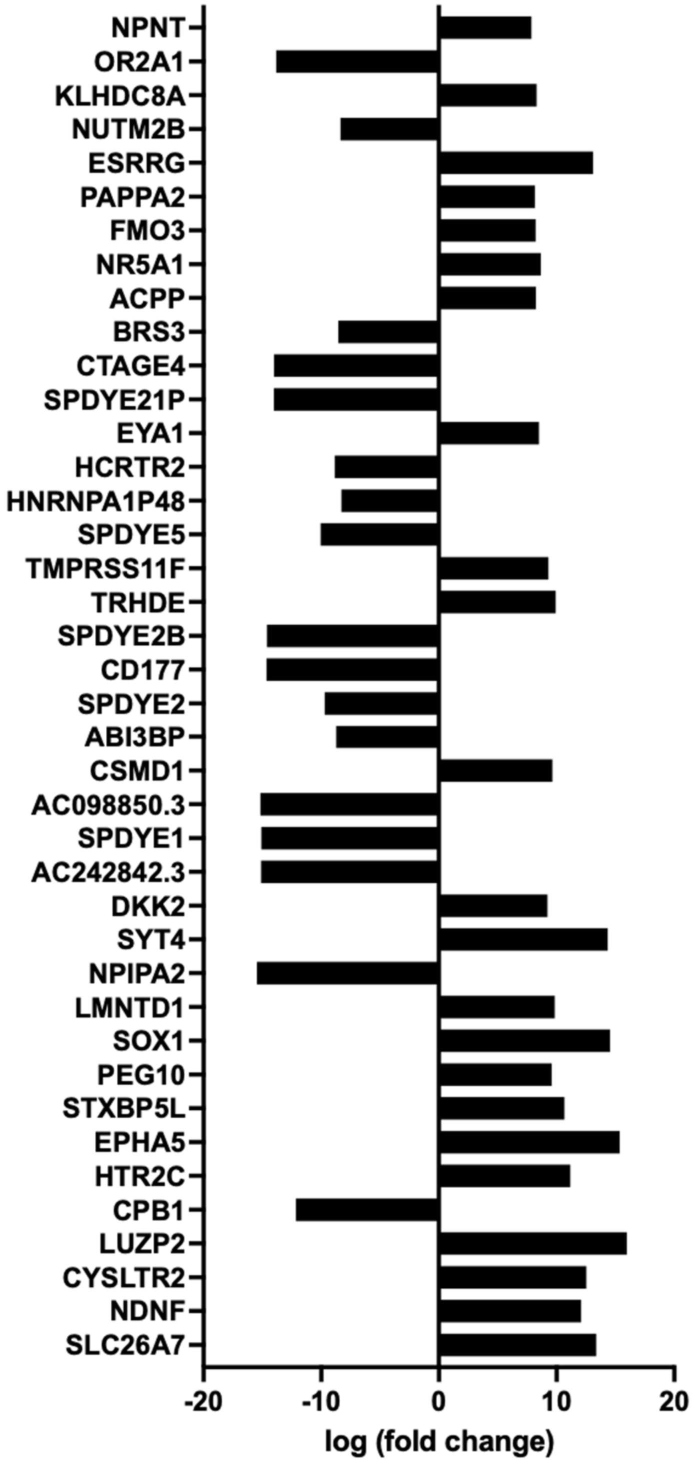

Supplement: Supplementary file 1 [file biomedicines-12-01709-s001.zip › biomedicines-3051281-supplementary.pdf]
